# Epigenetic Regulation of CDH1 Is Altered after HOXB7-Silencing in MDA-MB-468 Triple-Negative Breast Cancer Cells

**DOI:** 10.3390/genes12101575

**Published:** 2021-10-03

**Authors:** Ana Paço, Joana Leitão-Castro, Renata Freitas

**Affiliations:** 1 I3S—Institute for Innovation & Health Research, University of Porto, 4200-135 Porto, Portugal; anaisapaco@gmail.com (A.P.); joanaleitaocastro@gmail.com (J.L.-C.); 2 ICBAS—Institute of Biomedical Sciences Abel Salazar, University of Porto, 4050-313 Porto, Portugal

**Keywords:** HOXB7, breast cancer, triple-negative, MDA-MB-468, methylation

## Abstract

HOXB7 is often overexpressed in breast cancer cells and found to relate to poor prognosis. The search for the HOXB7 targets, as a transcription factor, has led to molecules involved in regulating cell proliferation, migration, invasion, and processes such as angiogenesis and therapy resistance. However, the specific targets affected by the deregulation of HOXB7 in breast cancer remain largely unknown in most molecular sub-types, such as triple-negative breast cancers (TNBC). To unveil the molecular basis behind these aggressive and often untreatable cancers, here we explored the contribution of HOXB7 deregulation for their aggressiveness. To this end, HOXB7 was silenced in TNBC Basal A cells MDA-MB-468, and the phenotype, gene/protein expression, and methylation profile of putative targets were analyzed. Lower migration and invasion rates were detected in HOXB7-silenced cells in comparison with the controls. In addition, these cells expressed more CDH1 and less DNMT3B, and the promoter methylation status of CDH1 diminished. Our data suggest that the HOXB7 transcription factor may act on TNBC Basal A cells by controlling CDH1 epigenetic regulation. This may occur indirectly through the up-regulation of DNMT3B, which then controls DNA methylation of the CDH1 promoter. Thus, future approaches interfering with HOXB7 regulation may be promising therapeutic strategies in TNBC treatment.

## 1. Introduction

HOX transcription factors act as transcription factors that regulate the coordinated expression of multiple genes crucial for embryonic development and cellular homeostasis during adulthood [1]. These proteins are encoded by the HOX clusters, first discovered in *Drosophila* and then found to control also vertebrates’ segment identity during development through regulation of cellular proliferation, differentiation, and death [2]. Humans display four HOX clusters (A, B, C, D) containing a total of 39 genes, located in chromosomes 7, 17, 2, and 12, respectively. 

The first connections found between aberrant HOX gene expression and malignant transformation were detected in leukemia [3]. However, it is now established that HOX genes present altered expression levels in a variety of solid tumors and derivative cell lines [4,5,6,7]. Several in vitro and in vivo studies have shown that HOX gene deregulation can promote cellular transformation and tumor progression, and some others have pointed out that deregulation of particular HOX proteins can also promote tumor suppressor effects [4,5,6,7].

The HOXB7 protein, encoded from the human HOXB cluster, has been implicated in hematopoietic differentiation, in lymphoid development, and in the development and malignant transformation of the mammary glands [8,9]. First, this protein was proposed to act as a trans-activator in breast cancer cells, increasing the expression of cellular proto-oncogenes [10,11]. Then, its overexpression was reported in a variety of breast cancer cell lines and primary tumors [12,13,14]. In addition, microarray analyses of dissected epithelial cells from bone metastasis revealed that HOXB7 overexpression was threefold higher than in primary breast carcinomas and eighteen-fold higher than in normal breasts [15]. In some cases, higher expression levels of HOXB7 were found to correlate with poorer disease-free survival in ERα+ breast cancer patients on adjuvant tamoxifen therapy [16].

In vitro studies using SkBr3 breast cancer cells provided the first evidence on how HOXB7 deregulation may affect breast cancer progression. These cells are representative of the HER2-positive molecular subtype, and in that genetic background, the induced transduction of HOXB7 leads to bFGF and VEGF expression, among many other growth and angiogenesis factors [10]. It also stimulates tumor development and vascularization in vivo, as shown in nude mice xenografts [17]. Interestingly, the induced overexpression of the mice counterpart (HOXB7) suggests a dual role in HER2/neu-induced mammary tumors: it delays tumor onset but promotes metastatic tumor progression. A link was also found between HOXB7 function and resistance to ionizing radiation in transformed mammary epithelial cells, MCF10A, due to regulation of proteins responsible for DNA double-strand break repair (Ku70, Ku80, DNA-PK(cs)) [18]. Moreover, HOXB7 overexpression in MCF10A cells induces epithelial-to-mesenchymal transition, which reinforces the importance attributed to HOXB7 expression in the formation of metastasis during breast neoplasia [15]. In another breast cancer cell line, MCF7, which represents the Luminal A molecular subtype, HOXB7 overexpression renders cells resistant to tamoxifen via cross-talk between receptor tyrosine kinases and ERα signaling [19]. In fact, the treatment of MCF-7 cells with tamoxifen results in increasing levels of HOXB7 expression, along with EGFR and EGFR ligands that lead to tamoxifen resistance. Thus, HOXB7 overexpression seems to potentiate proliferation and invasion in MCF7 cells, and its antagonism seems to be crucial to circumvent tamoxifen resistance. In addition, Ma and colleagues suggested that HOXB7 transiently silencing in MCF7 cells leads to inhibition of migration and invasion [20]. 

The role of HOXB7 was also addressed in triple-negative breast cancer (TNBC) basal B cell line MDA-MB-231 [21]. These cells, which are derived from metastatic sites (pleural infusion), present some degree of HOXB7 overexpression [14]. Here, the HOXB7 protein was proposed to bind to downstream targets such as CDH1 and DNMT3b, among others [21]. CDH1 encodes a cell adhesion molecule (E-cadherin), which is present in the adherent’s junctions of epithelial cells, preventing their transition to a mesenchymal migratory state [22]. Thus, it acts as a tumor suppressor gene that, when silenced or mutated, leads to the progression of a variety of cancers, such as gastric, colorectal [23], prostate [24], ovarian, and breast cancer [25]. The cause of CDH1 loss-of-function in cancer is attributed often to its transcriptional silencing via promoter hypermethylation [26]. However, the molecular pathways underlying CDH1 epigenetic silencing are still poorly explored. 

Taking into consideration the first studies addressing the role of HOXB7 in TNBC, here we suggest that CDH1 expression might be regulated by epigenetic mechanisms in these carcinomas, and we propose that HOXB7 overexpression may affect CDH1 expression directly but also indirectly through regulation of the methyltransferase DNMT3b. Keshet and colleagues suggested that the epigenetic silencing of tumor suppressor genes could occur randomly or through a targeted pathway initiated by an oncogene [27], which in our case might be HOXB7. Moreover, another HOXB protein, HOXB3, was found to interact directly with DNMT3B, leading to the up-regulation of the tumor suppressor gene RASSF1A [28]. Given that HOX genes from the same cluster have conserved domains and thereby may play synergistic or complementary functions, DNMT3B may indeed be also a target of the HOXB7 protein, as demonstrated for HOXB3. 

To explore the hypothesis that HOXB7 overexpression may affect CDH1 expression in TNBC cells directly or indirectly through an effect on DNMT3b transcription, we transiently silenced HOXB7 in another triple-negative cellular model, MDA-MB-468. However, that belongs to the basal A types, known to have much higher expression of CDH1 and more prominent overexpression of HOXB7 than the MDA-MB-231 cells [21]. This cell line is also derived from metastatic sites (pleural infusion) and presents an altered expression of p53, PTEN, and EGFR [29]. Our results suggest that HOXB7 silencing in these cell lines indeed impacts CDH1 and DNMT3B expression and causes a reduction in the promoter methylation status of CDH1. Taken together, these findings suggest that the HOXB7 transcription factor interferes with mechanisms that are involved in the epigenetic regulation of CDH1. This knowledge might be crucial to develop novel therapeutic approaches in TNBC designed to target the regulation of HOXB7 expression.

## 2. Materials and Methods

### 2.1. Cell Culture

Cell lines MDA-MB-468 and MDA-MB-231 were authenticated by the Genomics Scientific Platform at i3S using the PowerPlex16 HS System (Promega Corporation, Madison, WI, USA), and detection of the amplified fragments was performed with automated capillary electrophoresis using the 3130 Genetic Analyzer (Applied Biosystems, Waltham, MA, USA). The assignment of genotypes was made in GeneMapper software v5.0 (Applied Biosystems). The cells were cultured in Dulbecco’s modified Eagle’s medium (DMEM) supplemented with 10% fetal bovine serum and with 1% antibiotic solution (penicillin–streptomycin; Gibco Thermo Fisher Scientific, Waltham, MA, USA). Primary epithelial cell culture PCS-600-010, from ATCC, was used as a control, and its maintenance was performed according to the supplier’s instructions. The cells were cultured in a humidified atmosphere with 5% CO_2_ at 37 °C until reaching 80–90% of confluence.

### 2.2. HOXB7 Knockdown by RNA Interference and Phenotypic Characterizations

To generate HOXB7-silenced cells and controls, MDA-MB-468 and MDA-MB-231 were transfected with 10 μM of a SiRNA for HOXB7 (SiHOXB7) targeting the sequence 3′-ugaagcagaggaggaagaggaagag-5′ (Hs_HOXB7_4 FlexiTube siRNA, Qiagen, Hilden, Germany, Thermo Fisher Scientific, USA). SiRNA (SiNEG) was used as negative control (siRNA neg, Qiagen ID: 1022076) together with HiPerFect transfection reagent (QIAGEN, Thermo Fisher Scientific, USA). At three time points post-transfection, cells were processed for gene/protein expression assays: 24, 48, and 72 h, and experiments were replicated at least three times. The analyses of growth rate, actin filament organization, migration, and invasion abilities were performed as described by Borges and colleagues 48 h post-transfection [30]. In the wound-healing assays, the extent of wound closure was measured using the MRI wound healing tool from ImageJ software [31]. In the invasion assays, ten random fields from each membrane were photographed using the Zoe fluorescent cell imager (Bio-Rad, Amadora, Portugal) and the cell nuclei were counted using the Analyze Particles tool from ImageJ software [31].

### 2.3. Gene Expression Analyses 

The total RNA of the MDA-MB-231 and MDA-MB-468 transfected with siHOXB7 and siNEG was extracted using the AllPrep DNA/RNA/Protein Mini Kit (QIAGEN). DNA contamination was removed using DNase I (Roche Diagnostics, Basel, Switzerland) digestion, followed by RNA cleanup using RNeasy spin columns of the AllPrep DNA/RNA/Protein Mini Kit. A unit of 1 μg of total RNA was subjected to reverse transcription for cDNA synthesis using the High-Capacity RNA-to-cDNA™ Kit (Applied Biosystems, Thermo Fisher Scientific, USA). Quantitative real-time PCR experiments were performed using the TaqMan approach (Life Technologies Applied Biosystems, Thermo Fisher Scientific, USA). For that, TaqMan assays were performed for the target genes HOXB7, CDH1 (E-cadherin), and DNMT3B (DNA (cytosine-5-)-methyltransferase 3 beta) (Life Technologies Applied Biosystems, Hs04187556_m1, Hs01023894_m1, Hs00171876_m1). In the TaqMan assay, the GAPDH gene (glyceraldehyde-3-phosphate dehydrogenate) was used as a reference (Life Technologies Applied Biosystems, 4333764T). The 20 μL PCR reactions included 500 ng of the total of cDNA, 1 μL of the primer/probe assay mixture, 10 μL of PCR Master Mix (Life Technologies Applied Biosystems), and 8.5 μL of DEPC-treated water. This experiment was carried out in the CFX qPCR system (Bio-Rad, Portugal). All the experiments were performed in triplicate. The analyses of the Quantitative real-time PCR results were performed using 2^−ΔΔCT^ analyses.

### 2.4. Protein Expression Analyses

Protein extracts were prepared as previously described in Sousa et al. [32]. Western blots were probed with the HOXB7 rabbit polyclonal antibody (Origen, TA343468), the β-Tubulin mouse monoclonal antibody (Santa Cruz Biotech, 5-1-2), the DNMT3B rabbit polyclonal antibody (Fisher Scientific, PA1-884), or the CDH1 rabbit monoclonal antibody (Cell Signaling, 24E10). All the experiments were performed in triplicate, and the quantification of the blot signals was performed using the Image Lab software (version 6.0).

### 2.5. DNA Analyses 

DNA was extracted from HOXB7-silenced cells and controls using AllPrep DNA/RNA/Protein Mini Kit (QIAGEN, Thermo Fisher Scientific, USA) according to the manufacturer’s instructions. Then, PCRs were performed using the following primers: HOXB7Fw 5′-CTTTTTGGTGTAAATCTGGA-3′, HOXB7Rv 5′-CTTTAGATACACACAGAATGT-3′, CDH1Fw 5′-ATGGGCCCTTGGAGCCGCAG-3′, CDH1Rv 5′-CTAGTCGTCCTCGCCGCCTC-3′, DNMT3BFw 5′-GAAGGGAGAGAGCAAACAAA-3′ and DNMT3BRv 5′-AATACTGATTTTAATTAAAC-3′. These reactions had, for a final volume of 50 μL, 1× reaction buffer, 20 ng of total DNA, 0.2 mM of each dNTP, 15 pmol of each primer, 1.5 mM of MgSO_4_, and 0.02 U of the high fidelity KOD Hot Start DNA polymerase (Merck Millipore, Darmstadt, Germany). The amplification program was 2 min of initial denaturation at 95 °C, 30 cycles of 10 s at 95 °C, 10 s at 65 °C, and 85 s at 70 °C. The PCR products were purified using the GFX DNA purification Kit (GE Healthcare, Little Chalfont, UK), cloned in pGEM-T (Promega, Madison, WI, USA), and six clones were sequenced. 

### 2.6. 5-Aza-2′-Deoxycytidine Treatment

MDA-MB-231 cells were treated with DNA-hypomethylating compound 5-Aza-2′-deoxycytidine for 48 h. Stock solutions were made in DMSO and diluted to concentrations of 1 μM and 5 μM in Dulbecco’s modified Eagle’s medium (DMEM) supplemented with 10% fetal bovine serum and 1% antibiotic solution (penicillin–streptomycin; Gibco Life Technologies). DMSO was used as vehicle control in the concentration of 5 μM. All the experiments were performed in triplicate. Upon the 48 h incubation period, cells were processed for gene expression analyses.

### 2.7. Methylation Analyses

DNA extracted from HOXB7-silenced cells and controls were bisulfite converted using the EpiTect Bisulfite Kit according to the manufacturer’s instructions (QIAGEN). The converted DNA was then used in PCR reactions, allowing the amplification of a 408 bp fragment from the CDH1 promoter, with 22 CpG sites. The 408 bp of the CDH1 promoter sequence were amplified between the positions 648 and 1056 bp of this sequence using primer forward 5′-GTATTTTAGTTTGGGTGAAAGAG-3′ and primers reverse 5′-CAAACTCACAAATACTTTACAATTC-3′. The obtained PCR fragments were cloned into a pGEM-T vector (Promega, (Promega, Madison, USA)), and for each cell line, ten clones were sequenced using the universal primer M13. The methylation status of the CDH1 promoter in HOXB7-silenced cells and controls was evaluated using the BISMA-Bisulfite sequencing DNA Methylation Analysis program.

### 2.8. Statistical Analysis

The statistical differences between two variables (siNEG and siHOXB7) were determined using Test-*t* paired samples. The statistical differences between three variables (24 h, 48 h, 72 h) were evaluated using the post hoc Tukey test (*p* < 0.05, where different lettering corresponds to statistical differences (a, no differences; b, differences)). Statistical analyses were carried out on SPSS V.21 software (SPSS Inc., Chicago, IL, USA).

## 3. Results

### 3.1. HOXB7-Silencing in Triple-Negative Breast Cancer Cells

**Figure 1:**
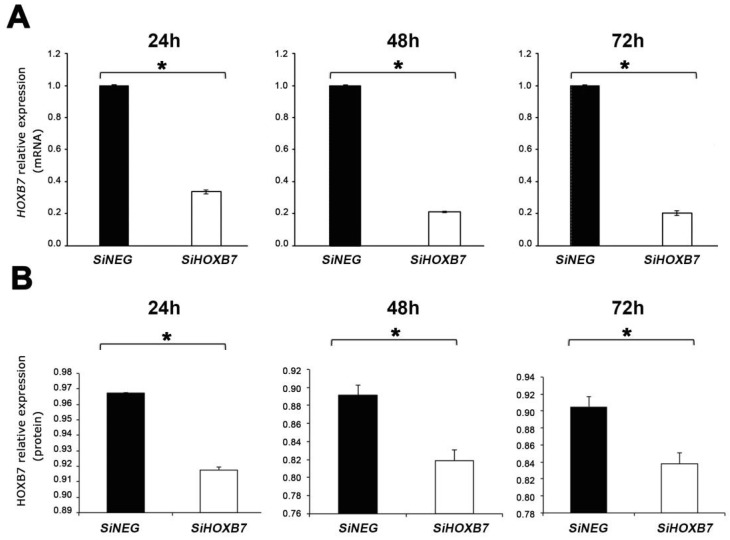
HOXB7 expression in MDA-MB-468 cells transfected with siHOXB7 and control siNEG evaluated by RT-qPCR (**A**) and western-blot (**B**) analyses at 24, 48, and 72 h (h) post-transfection (three replicates). Statistical analyses and significant differences are indicated with asterisks based on Test-*t* analyses (*, *p* < 0.001). Expression differences between time points were compared using the post hoc Tukey test. The HOXB7 mRNA levels were approximately 70% lower in HOXB7-silenced cells than in the controls, and the protein levels were also lower in the HOXB7-silenced cells just detectable at 24 h and 48 h post-transfection. In the western-blot analyses, β-tubulin was used as the reference protein.

HOXB7 is overexpressed in all breast cancer (BC) cell lines studied, including in the MDA-MB-468 (Supplementary Figure S1), which is representative of the triple-negative molecular subtype [5,14]. Considering the lack of knowledge on the putative targets of the HOXB7 protein in this genetic background, functional assays were conducted using MDA-MB-468 aiming to further explore the mechanistic effect of HOXB7 upregulation in triple-negative BC cells. To this end, these cells were transfected with siHOXB7 and siNEG as a control, and then the expression levels of HOXB7 were evaluated by RT-qPCR and western-blot analyses at 24, 48, and 72 h post-transfection. Reduced mRNA and protein levels were detected at these time points (Figure 1A,B).

**Figure 2:**
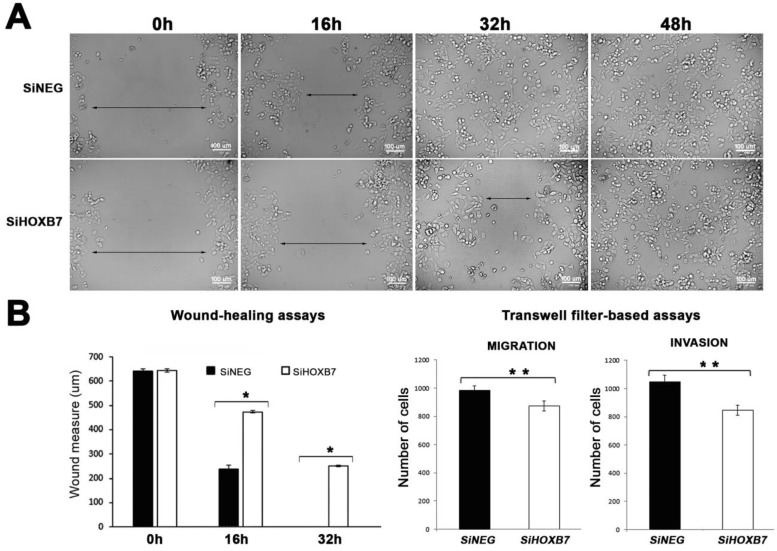
Analyses of cell migration and invasion in MDA-MB-468 cells transfected with siHOXB7 and control siNEG. Wound-healing assays suggest reduced capacity to migrate in HOXB7-silenced cells, detectable after 16h post-transfection (**A**). Transwell filter-based assays also point to lower migration and invasion abilities in HOXB7-silenced cells than in the controls (**B**). Three independent biological replicates were used in the statistical analyses, and significant differences are indicated with asterisks (*, *p* < 0.001; **, *p* < 0.05), based on *t*-test analyses.

The impact of HOXB7 silencing was then further evaluated addressing changes in the migration and invasion abilities (Figure 2A). Performing wound-healing assays, a lower capacity to migrate was detected in the HOXB7-silenced cells in comparison with the controls after 16 h post-transfection. The migration ability of these cells was also measured using transwell filter-based assays (Figure 2B), which suggested that HOXB7-silenced cells have a reduced ability to migrate compared to the controls. No alterations were detected in apoptosis or in the organization of F-actin filaments between HOXB7-silenced cells and controls (Supplementary Figure S2).

### 3.2. Impact of HOXB7-Silencing in Putative Downstream Targets

**Figure 3:**
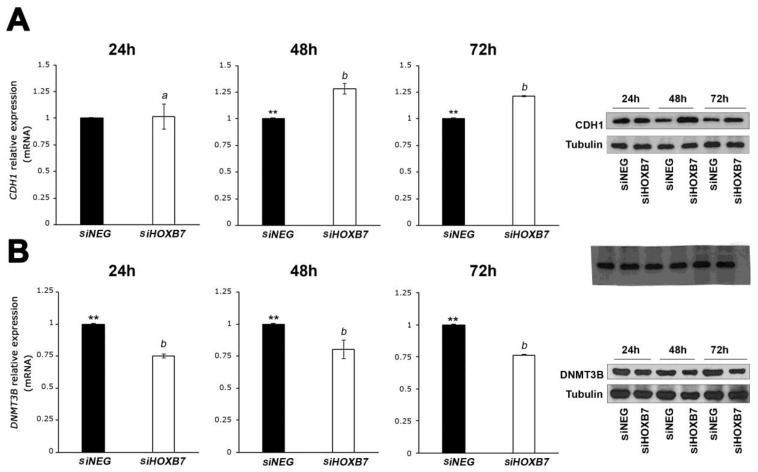
Expression analyses of CDH1 (**A**) and DNMT3B (**B**) in MDA-MB-468 cells transfected with siHOXB7 and siNEG analyzed by RT-qPCR and western blots at three time points post-transfection. Three independent biological replicates were used in the statistical analyses comparing two variants (siNEG and siHOXB7) and the significant differences indicated with asterisks based on the analyses using *t*-test. The differences in the expression measures at the three time points were compared using the post hoc Tukey test. CDH1 expression was significantly higher in siHOXB7-transfected cells than in controls at 48 h and 72 h (**, *p* < 0.05). Comparing the three time points, the expression of CDH1 is significantly different at 24 h in these cells (*a*) in comparison with 48 h and 72 h (*b*). No significant differences in CDH1 expression were detected between 48 h and 72 h. DNMT3B expression was significantly lower in HOXB7-silenced cells than in the controls in the three time points analyzed, and no significant differences were detected in the expression between these time points (*b*). In the western-blot analyses, β-tubulin was used as the reference protein. *a*: significant expression differences between time-points in the siHOXB7 cells evaluated with post hoc Tukey test; *b*: no significant expression differences between time-points in the siHOXB7 cells evaluated with post hoc Tukey test.

Taking into account the changes in migration and invasion in HOXB7-silenced cells, we next evaluated the expression of E-cadherin (CDH1) in the cells transfected with siHOXB7 and control siNEG by RT-qPCR and western-blot analyses. This adherent junction protein may play an active role in the epithelial-to-mesenchymal transition (EMT) potentially involved in BC cell migration and metastasis, and its downregulation has been associated with poor prognosis [26]. An up-regulation of CDH1 in the HOXB7-silenced cells was detectable at 48 h and 72 h post-transcription (Figure 3A), a modification that may have contributed to the lower migration and invasion abilities detected in these cells (Figure 2). In addition, a reduction in vimentin expression (VIM) was detected after the transfection with siHOXB7 in MDA-MB-468 cells (Supplementary Figure S3), which also relates to the results obtained concerning cell migration and invasion after HOXB7-silencing. When upregulated, this mesenchymal marker promotes the EMT by potentiating the migration and invasion of BC cells [33].

Taking into account that CDH1 expression might be epigenetically regulated by promoter methylation [34], the next question was if the effect of the HOXB7 protein upon CDH1 expression could be indirect, by controlling first the expression of methyltransferases, such as DNMT3B. A similar process was proposed for HOXB3, which participates in the epigenetic silencing of the tumor suppressor gene RASSF1A through the induction of DNMT3B expression [28]. To explore this hypothesis, the expression of DNMT3B was evaluated in HOXB7-silenced cells and controls. Downregulation was detected 48 h after the HOXB7-silencing (Figure 3B).

### 3.3. Impact of HOXB7-Silencing in the Methylation Profile of the E-Cadherin Promoter in Triple-Negative Breast Cancer Cells

**Figure 4:**
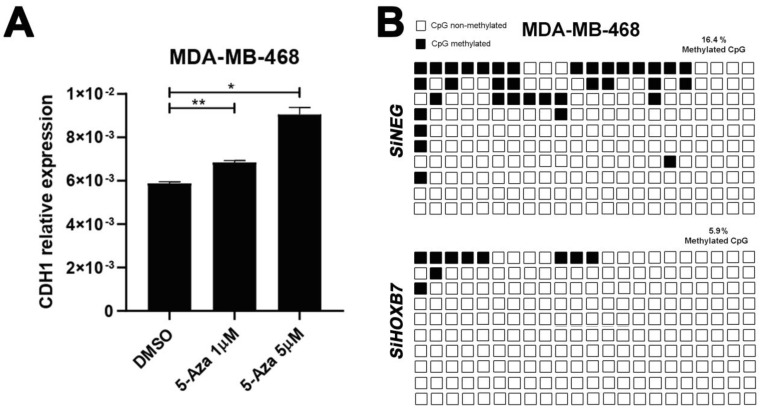
Influence of HOXB7 overexpression in CDH1 regulation in MDA-MB-468 triple-negative breast cancer cells. (**A**) Expression analyses of CDH1 in 5-Aza-2′deoxycytidine treated cells analyzed by RT-qPCR. Three independent biological replicates were used in the statistical analyses, and significant differences were indicated with asterisks based on the analyses using *t*-test (**, *p* < 0.05, *, *p* < 0.001). (**B**) Methylation status of the CDH1 promoter in HOXB7-silenced cells and controls. Bisulfite sequencing analyses revealed lower CDH1 promoter methylation in cells transfected with siHOXB7 than in controls transfected with siNEG. CpGs regions are identified by squares: black squares represent methylated cytosine and white squares represent unmethylated cytosine. Each row represents a single clone.

We first investigated if CDH1 expression is modulated by methylation in the triple-negative genetic background. To this end, we exposed MDA-MB-468 cells to two distinct concentrations of a DNA-hypomethylating agent, 5-Aza-2′-deoxycytidine (5-Aza), and found, subsequently, an increase in the CDH1 expression in comparison with controls (Figure 4A). These data are in agreement with previous reports suggesting that CDH1 is a methylation-silenced gene in these cells [35]. We then investigate whether HOXB7 overexpression may have an impact on the methylation of E-cadherin in MDA-MB-468 cells, as reported recently for other triple-negative breast cancer cells [21]. To this end, we characterized the CDH1 methylation status in HOXB7-silenced cells and controls using bisulfite conversion followed by sequencing analyses. Our results suggest that 48 h after the HOXB7-silencing, the CDH1 promoter appears less methylated in comparison with the controls, dropping from 16.4% in cells transfected with SiNEG to 5.9% in cells transfected with SiHOXB7 (Figure 4B). We observed similar data for MDA-MB-231 (Supplementary Figure S4), despite the methylation profile in these two cell lines being very distinct.

## 4. Discussion

Triple-negative BC represents 10–20% of all breast cancer cases and is not responsive to hormone therapy, and the chemotherapy is not efficient at long times and presents the worst prognosis [36]. In this work, we aimed to contribute to the understanding of the molecular pathways by which HOXB7 affects Triple-negative BC cells, obtaining knowledge that may contribute to developing future novel therapy approaches. To this end, we investigated processes that could be affected by the deregulation of HOXB7 in MDA-MB-468 cells, which are derived from a metastatic site and without any available information regarding putative downstream targets. The overexpression of HOXB7, compared to normal epithelial breast cells, was confirmed using western-blot analyses, which is in agreement with previous work using distinct approaches [5,14]. 

When HOXB7 was silenced in MDA-MB-468 cells, using specific siRNAs, a less aggressive phenotype was detected together with reduced ability to migrate and invade. To understand the molecular basis behind this, we evaluated the expression of E-cadherin, encoded by the tumor suppressor gene CDH1. The expression levels of E-cadherin, together with P-cadherin, strongly correlate with high-grade breast carcinomas, biologically aggressiveness, and poor patient survival [37]. This protein is essential to maintain the cellular epithelial state, keeping the cell-to-cell adhesion and preventing cell motility [38]. Our data showed that with the silencing of HOXB7 in MDA-MB-468 cells, the expression of CDH1 increased. Recent functional assays performed in MDA-MB-231 cells revealed enriched interactions between the HOXB7 protein and CDH1 [21]. Thus, we hypothesize that HOXB7 deregulation may affect CDH1 expression in multiple triple-negative BC cells. 

Our data also revealed that, while the expression of CDH1 increased in the HOXB7-silenced cells, the expression of DNMT3b diminished. Interestingly, this gene was also found to be a putative target of HOXB7 in another type of triple-negative BC cell [21]. This suggests that, in this genetic background, particular levels of HOXB7 may have a positive impact on DNMT3B transcription and, in this way, may contribute directly or indirectly to regulate epigenetically the expression of CDH1. In addition, we indeed found that the methylation of the CDH1 promoter was reduced in MDA-MB-468 cells, in which HOXB7-silencing was induced, in comparison with the controls, which probably favored the detected CDH1 overexpression. The methylation of the CDH1 promoter dropped more than 50% in comparison with the control. Thus, our data seem to uncover a novel role for HOXB7 in triple-negative BC cells, which may relate to the epigenetic control of E-cadherin. 

Overall, the obtained data produced knowledge to the development of novel therapeutic strategies to treat triple-negative breast cancers. Future approaches rooted in the control of HOXB7 expression could be a promising strategy to treat TNBC.

## Data Availability

Not applicable.

## Supplementary Materials

The following are available online at https://www.mdpi.com/article/10.3390/genes12101575/s1: Figure S1: Expression analyses of Vimentin (VIM) in MDA-MB-468 cells transfected with si-HOXB7 or siNEG. Characterization of the apoptosis and the F-actin filaments in HOXB7-silenced cells and controls (MDA-MB-468); Figure S2: Methylation status of the CDH1 promoter in MDA-MB-231 HOXB7-silenced cells and controls; Figure S3 Expression analyses of Vimentin (VIM) in MDA-MB-468 cells transfected with SiHOXB7 or SiNEG, suggesting a decrease when HOXB7 is silenced; Figure S4: Influence of HOXB7 overexpression in CDH1 regulation in MDAMB-231 Triple-negative breast cancer cells.
