# Supplementary material for: Epigenetic Regulation of CDH1 Is Altered after HOXB7-Silencing in MDA-MB-468 Triple-Negative Breast Cancer Cells
Source: Genes (Basel). 2021 Oct 3;12(10):1575. doi: 10.3390/genes12101575 (PMC8535730; doi:10.3390/genes12101575)
Supplement: Supplementary file 1 [file genes-12-01575-s001.zip › genes-1377963-supplementary.pdf]

Supplementary Data

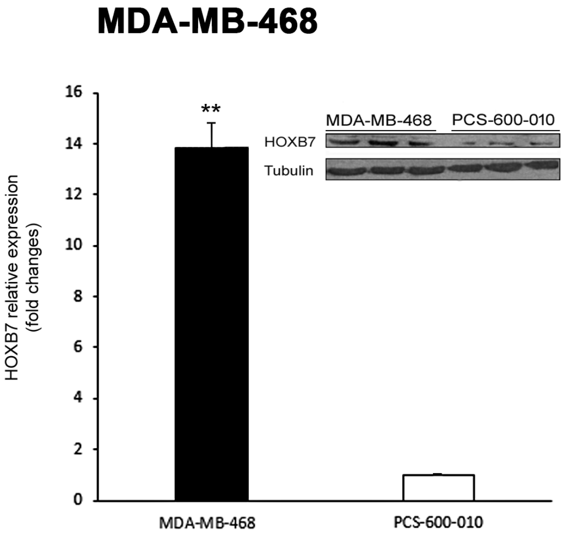

Supplementary Figure S1: Expression analyses of HOXB7 in breast cancer cells MDA-MB-468 and in normal mammary cells PCS600-010. HOXB7 expression results were normalized with the expression of β-tubulin and presented as the mean and standard deviations of three independent biological replicates. All the differences are statistically significant (**) following analyses using Test-T with a P value <0.001.

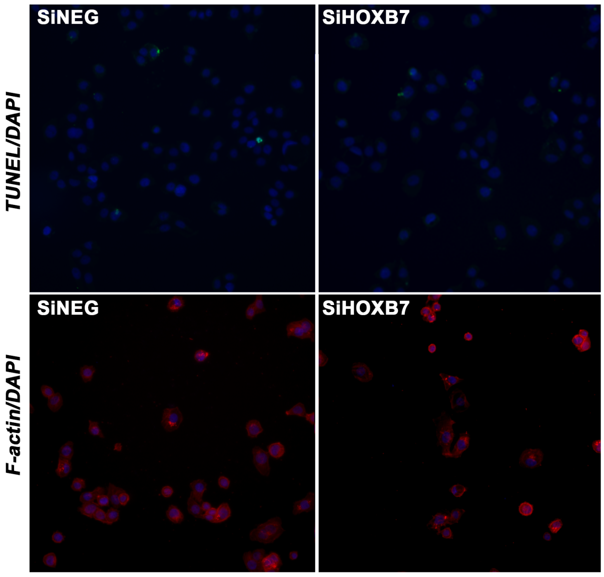

Supplementary Figure S2: Characterization of the apoptosis and the F-actin filaments in *HOXB7*-silenced cells and controls (MDA-MB-468). Green staining indicates apoptotic cells using the TUNEL method. No differences have been detected between the *HOXB7*-silenced cells and controls. Red staining marks the F-actin filaments detected using Phalloidin-iFluor 555 reagent (Abcam). No differences have been detected between the *HOXB7*-silenced cells and controls.

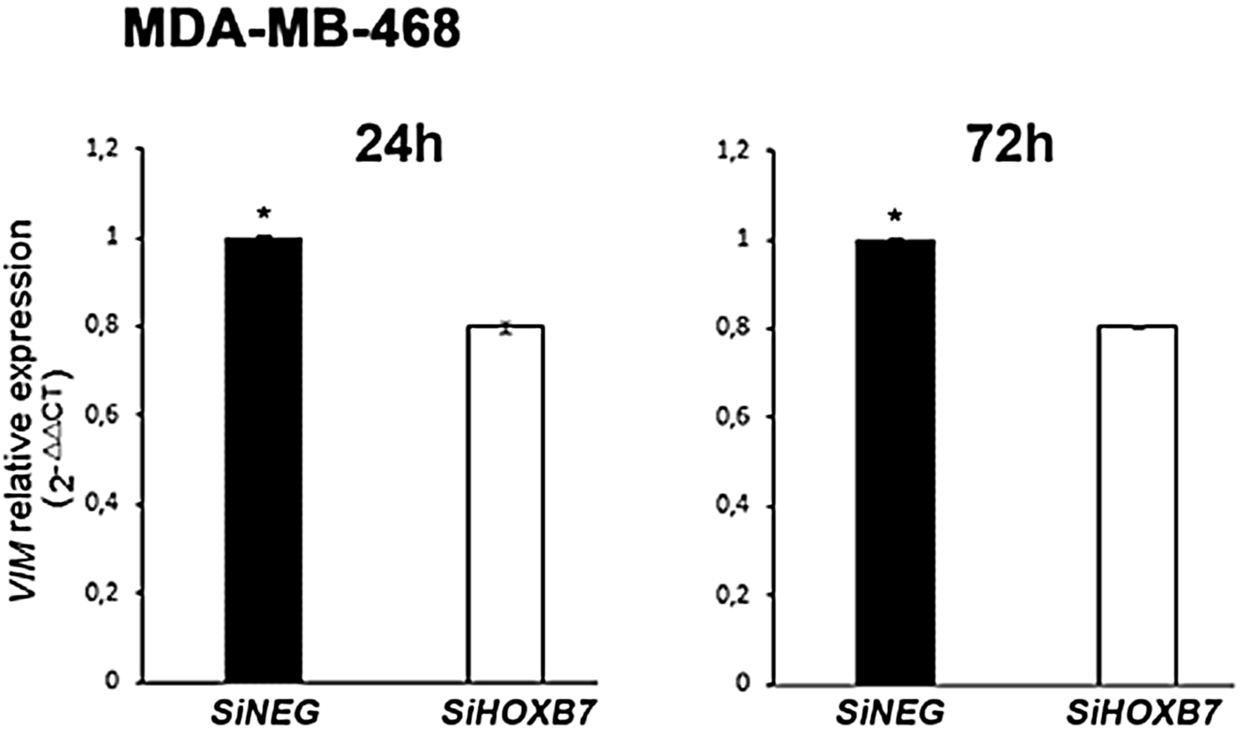

Supplementary Figure S3: Expression analyses of *Vimentin* (*VIM*) in MDA-MB-468 cells transfected with SiHOXB7 or SiNEG, suggesting a decrease when *HOXB7* is silenced. Statistically significant differences are indicated with an asterisk (*, p>0,05) and were calculated using T-Test. The differences in the expression measure at the two time points were compared using the post hoc Tukey test and no differences were identified in the expression of *VIM* expression in cells transfected with SiHOXB7 at 24h or 72h.

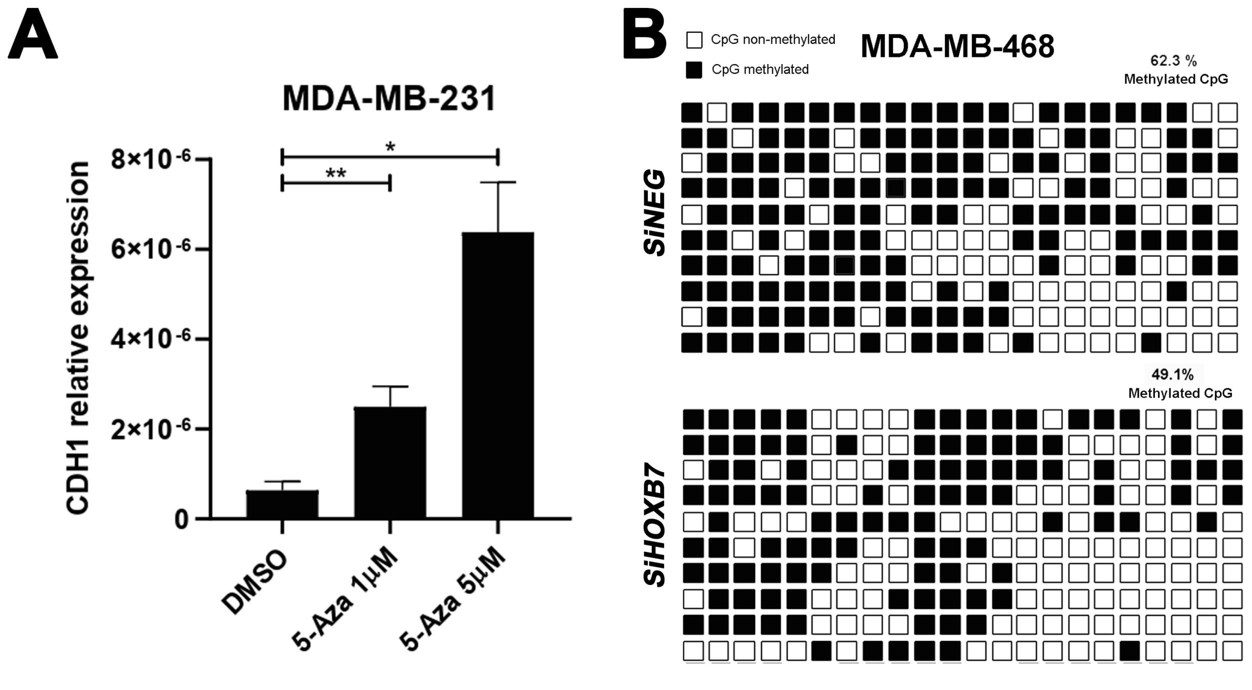

Supplementary Figure S4: Influence of *HOXB7* overexpression in *CDH1* regulation in MDA-MB-231 Triple-negative breast cancer cells. (A) Expression analyses of *CDH1* in 5-Aza-2'deoxycytidine treated cells analyzed by RT-qPCR. Three independent biological replicates

were used in the statistical analyses and significant differences were indicated with asterisks based on the analyses using T-test (*, p <0.05, **, p <0.001). (B) Methylation status of the *CDH1* promoter in *HOXB7*-silenced cells and controls. Bisulfite sequencing analyses revealed lower CDH1 promoter methylation in cells transfected with siHOXB7 than in controls transfected with siNEG. CpGs regions are identified by squares: black squares represent methylated cytosine and white squares unmethylated cytosine. Each row represents a single clone.
